# Educational Attainment, Health Status, and Program Outcomes in Latino Adults With Arthritis Participating in a Walking Program

**DOI:** 10.5888/pcd15.180129

**Published:** 2018-10-18

**Authors:** Leigha Vilen, Rebecca J. Cleveland, Leigh F. Callahan

**Affiliations:** 1 Thurston Arthritis Research Center, School of Medicine, University of North Carolina at Chapel Hill, Chapel Hill, North Carolina; 2 Division of Rheumatology and Immunology, Department of Medicine, University of North Carolina at Chapel Hill, Chapel Hill, North Carolina; 3 Departments of Social Medicine and Orthopaedics, University of North Carolina at Chapel Hill, Chapel Hill, North Carolina

## Abstract

**Introduction:**

Latinos are disproportionately likely to lack a high school diploma, compared with non-Hispanic whites, a trend associated with worse outcomes in arthritis and indicating a need for health interventions. *Camine Con Gusto* (CCG) is the Spanish version of the evidence-based Walk With Ease program for arthritis. This study compared baseline health status and examined differences in program efficacy and adherence among Latino adults with and without a high school diploma enrolled in a pre−post evaluation of CCG.

**Methods:**

CCG participants (n = 233) were classified into 2 groups: high school diploma or more (n = 129) and less than high school diploma (n = 104). We used logistic regression to estimate odds ratios (ORs) and 95% confidence intervals (CIs) for associations of education with measures of baseline health and program adherence. We computed effect sizes for the difference between education groups by using mean change scores for arthritis symptoms, physical function, and psychosocial variables.

**Results:**

The group without a high school diploma was more likely to report worse general health (OR = 2.40; 95% CI, 1.28–4.53) and lower levels of arthritis self-efficacy (OR = 1.95; 95% CI, 1.05–3.63) than the group with a high school diploma. CCG improved outcomes for both groups, with no significant between-group differences. The group without a high school diploma was less likely to read most of the program workbook (OR = 0.51; 95% CI, 0.27–0.97), but we found no significant differences in the amount of walking between the 2 groups.

**Conclusion:**

CCG was equally effective among Latinos with and without a high school diploma; however, education did affect participants’ engagement with the program workbook. Adaptation of interventions for Latinos should consider how information can best be conveyed to those with lower levels of formal education.

## Introduction

Arthritis is the most common cause of disability in the United States, affecting 54.4 million adults (1). Although Latinos have a lower rate of arthritis (15.4%) compared with non-Hispanic white adults (22.6%) and black adults (22.2%), they report higher levels of disability from arthritis (1,2).

Fewer years of formal education is associated with greater pain, disability, and activity limitations due to arthritis (3–5). Because 30.0% of Latinos living in the United States lack a high school diploma, compared with 7.1% of non-Hispanic whites, interventions for Latinos who have arthritis and low levels of education must be specially designed to be appropriate and efficacious (6).

The Walk With Ease program is a 6-week, evidence-based intervention for people with arthritis. Both the instructor-led group format and the workbook-guided self-directed format can reduce arthritis symptoms and increase physical performance up to 1 year after the program (7,8). With the exception of a secondary analysis of African American participants, at least 70% of all participants in evaluations of Walk With Ease have had at least a high school education (7–10). Although studies acknowledge education differences in preferred format (those with more education prefer the self-directed format), ours is the first study to evaluate possible differences in the program’s efficacy based on educational attainment (8,10).

This study was a secondary analysis of data from a pre–post evaluation of *Camine Con Gusto* (CCG), the Spanish translation and adaptation of Walk With Ease (11). The parent study, which evaluated CCG among 233 Latino adults with arthritis, found the program feasible, safe, acceptable, and efficacious at improving symptoms, physical function, and psychosocial measures in this population (11). The objectives of our study were to 1) compare baseline health status among participants with and without a high school diploma; and 2) examine arthritis outcomes and program adherence at follow-up to determine whether CCG’s efficacy differed between education groups.

## Methods

We analyzed data from baseline and 6-week follow-up assessments of 233 participants in the parent study of CCG (11), which was conducted from May through September 2014 in the area surrounding Chapel Hill, North Carolina. A bilingual team recruited participants from the rheumatology, gastrointestinal, geriatric, and internal medicine clinics in the University of North Carolina Hospitals Center for Latino Health (CELAH) program, a CELAH-sponsored health fair, the Mexican consulate, and 3 churches with Hispanic ministries. Recruitment is detailed elsewhere (11). Participants self-identified as Hispanic/Latino, were aged 21 years or older; reported arthritis, joint pain, or a diagnosis of arthritis by a health care professional; and were able to walk unassisted but were currently walking on average less than 150 minutes per week.

The parent study evaluated the CCG program only in the self-directed format. During recruitment, participants received a copy of the CCG workbook, which is written at a 6th-grade reading level and guides participants through developing a walking plan, getting started walking, overcoming barriers, and staying motivated. The workbook has 6 chapters and 183 pages, which participants are encouraged to read during the first 2 weeks and then reference throughout the 6-week program and beyond. The design of the CCG workbook is interactive, containing self-tests for participants to score pain, fatigue, and physical limitations and then suggesting different strategies for differing scores.

During recruitment, the bilingual team gave participants a brief explanation (approximately 5 minutes) of the program goals (walking 5 times/week for 30 minutes, or 150 minutes/week) and highlights of the workbook, including the self-test assessments, summary of information contained in each chapter, and warm-up and cool-down exercises. Participants completed baseline surveys in person or over the telephone and then completed a follow-up survey by mail or telephone after 6 weeks. All study procedures were approved by the Biomedical Institutional Review Board of the University of North Carolina at Chapel Hill.

### Measures

**Demographic characteristics.** We collected demographic information on race, age, education, sex, marital status, health status, comorbid conditions, and acculturation measures. Age was measured as a continuous variable based on date of birth. Education was assessed with the question, “What is the highest level of education you have finished in school? Please check.” Responses included grades 1 through 8, grades 9 through 11, high school graduate, some college, junior college diploma, college degree, some post-college work, or advanced degree. Body mass index (BMI, measured as weight in kilograms divided by weight in meters squared [kg/m^2^]) was calculated as a continuous measure by using self-reported height and weight. To measure number and type of comorbid conditions, participants self-reported nonarthritis conditions by using a 13-item checklist (cancer, fibromyalgia, glaucoma, emphysema, high blood pressure, heart disease, circulation problems, diabetes, stomach or intestinal disorders, osteoporosis, chronic liver or kidney disease, stroke, or depression). A short-form acculturation scale for Hispanics was used to record country of birth; parent’s county of birth; language spoken in childhood; language in which one thinks, reads, and writes; and language spoken at home and with friends (12). Because of the large proportion of respondents who were born in Mexico, we categorized answers to this question on country of birth as born in Mexico or born in another country. For the questions on language, the options were Spanish only, Spanish more than English, both English and Spanish, English more than Spanish, or English only.

**Primary outcome measures.** Primary outcome measures were physical function and arthritis symptoms. Physical function was assessed with the validated Spanish-modified Health Assessment Questionnaire scale, which measures difficulty in performing activities of daily living. The Health Assessment Questionnaire has 8 items with scores ranging from 0 (without any difficulty) to 3 (unable to do) (13). Arthritis symptoms (pain, fatigue, and stiffness) were assessed with visual numeric scales (range 0–10, with 0 being “none” and 10 being “pain/fatigue/stiffness as bad as can be”) (13). Pain and fatigue were assessed by using the validated Visual Numeric Pain scale and the Visual Numeric Fatigue scale, respectively (13,14). A visual numeric scale for stiffness was adapted from the Visual Analog Scale for stiffness to resemble the fatigue and pain scales (15,16).

**Secondary outcome measures.** Secondary outcomes were self-reported general health status and psychosocial measures. Self-reported general health status was measured by asking participants to rate their health status as excellent, very good, good, fair, or poor (17). Psychosocial measures were arthritis self-efficacy, measured by the 11-item short form Spanish-modified Arthritis Self-Efficacy Scale, and perceived helplessness, measured by the Spanish-modified helplessness subscale of the Rheumatology Attitudes Index (15,18,19).

**Program adherence measures.** Adherence with program objectives was assessed at 6 weeks by asking about walking behaviors and use of the CCG workbook. Participants were asked if they did any walking (yes, no), and if they did, how many days and minutes per week they walked (1 or 2 days [referent], 3 or 4 days, ≥5 days; <15 minutes [referent], 15–30 minutes, 30–45 minutes, >45 minutes). Workbook usage was assessed with the question, “How much of the *Camine Con Gusto* Workbook did you read?” (none [referent], a little, some [2 or 3 chapters], most [4 or 5 chapters], other ways [an open-response option, eg, “read about exercises in back of book”]). Although self-reported measurements of walking are subject to recall and social desirability bias and are not as reliable as objective measures, such as a wearable device, the parent study used self-reported measures. This choice was made because walking was not a primary outcome measure and because the study team interacted with participants only once and very briefly, precluding the provision of adequate instructions on how to use the device and collection of the device after the study (20).

### Analysis

We computed descriptive statistics for covariates at baseline, stratified by education (≥high school diploma, <high school diploma). High school diploma served as a binary cut-off point because roughly half of our participants were on each side of the cut-off. We tabulated continuous variables as mean and standard deviation and categorical variables as frequency and percentage. All tests were 2-sided and considered significant at *P* ≤ .05. All analyses were carried out in SAS software version 9.4 (SAS Institute Inc).

Baseline data were missing for BMI (21.0%) and marital status (13.7%). Analyses to explore whether data were missing at random did not find any significant associations between predictors and missingness of data. We used Markov Chain Monte Carlo methods with all covariates to impute missing covariate values in 40 data sets in SAS PROC MI. Markov Chain Monte Carlo methods are a class of algorithms that allow approximation of the posterior distribution by random sampling values from the distribution to fill in missing data.

Mixed effects logistic regression accounting for clustering by recruitment site was used to estimate the odds ratios (ORs) and 95% confidence intervals (CIs) for the association between education level with baseline values of binary health status measures, amount of workbook read, and days of walking. Cut-off points were selected for binary variables by using medians for all health status variables except visual numeric score scales and comorbidities. Medians for pain, fatigue, and stiffness visual numeric scale scores were all between 50 and 60, so 60 was conservatively selected as a cut-off point for all 3 scales to preserve consistency. For number of comorbidities, 2 comorbidities was used as a cut-off point because data for most participants clustered at 1 comorbidity, which was also the median.

We used multivariate linear mixed regression models with recruitment site as a random effects variable to calculate mean changes between baseline and 6-week follow-up scores, controlling for baseline outcome score and covariates. Mean change scores were used to estimate effect sizes, expressed as Cohen *d*, which was calculated by comparing the mean change scores from baseline to 6 weeks divided by the pooled standard deviation (21). We calculated mean change scores for all CCG participants for changes from baseline to follow-up, and we calculated effect sizes that compared mean change scores between those who did not complete high school and those who did. Covariates were age, sex, marital status, obesity, country of origin, primary language spoken, and number of comorbidities.

## Results

Educational attainment was generally low, with 44.2% (103 of 233) of study participants lacking a high school diploma (Table 1). Of participants who lacked a high school diploma, 95 of 103 (92.2%) reported having less than a 9th-grade education, and a significantly higher percentage were female (78.6%), born in Mexico (78.6%) and spoke only Spanish (79.6%) than in the group with a high school diploma (female, 76.9%; born in Mexico, 61.5%; speak only Spanish, 42.3%). A smaller percentage of the group without a high school diploma was obese (39.8% vs 48.5%); however, because a greater percentage (33%, n = 34) of this group had a very high BMI (≥40), compared with the percentage of the group with a high school diploma (15%, n = 20), mean BMI did not differ significantly between the 2 groups. Finally, a greater proportion of the group without a high school diploma were single (19.4% vs 16.2%), although the proportion of single participants was low in both groups.

**Table 1:** Baseline Characteristics of Participants in the *Camine Con Gusto* (Walk With Ease) Program^a^ Who Completed 6-Week Follow-Up, by Level of Education, North Carolina, May–September, 2014

Baseline Characteristic	All Participants (N = 233)	<High School Diploma (n = 103)	≥High School Diploma (n = 130)	*P* Value^b^
**Age, mean (SD), y**	47.0 (11.0)	48.1 (11.8)	46.1 (10.6)	.17
**Female, no. (%)**	181 (77.7)	81 (78.6)	100 (76.9)	.04
**Marital status, no. (%)**				
Single	41 (17.6)	20 (19.4)	21 (16.2)	<.001
Married	160 (68.7)	73 (70.9)	88 (67.7)	
Other	32 (13.7)	11 (10.7)	21 (16.0)	
**Body mass index, mean (SD), kg/m^2^**	30.2 (6.8)	30.0 (6.6)	30.3 (7.1)	.76
**Obese, no. (%)^c^**	104 (44.6)	41 (39.8)	63 (48.5)	<.001
**Acculturation, no. (%)**				
Born in Mexico	161 (69.1)	81 (78.6)	80 (61.5)	<.001
Speak Spanish only	137 (59.8)	82 (79.6)	55 (42.3)	<.001
**Self-reported health status**				
Fatigue score^d^, mean (SD)	48.9 (30.5)	52.8 (30.7)	45.7 (30.3)	.08
Pain score^d^, mean (SD)	57.5 (25.1)	62.1 (25.3)	53.9 (24.9)	.01
Stiffness score^d^, mean (SD)	47.5 (29.5)	49.3 (32.0)	46.2 (27.7)	.43
Arthritis Self-Efficacy Scale score, mean (SD)^e^	7.1 (2.2)	6.8 (2.2)	7.4 (2.2)	.03
Health Assessment Questionnaire score, mean (SD)^f^	0.5 (0.5)	0.5 (0.5)	0.4 (0.4)	.20
Rheumatology Attitudes Index score, mean (SD)^g^	1.4 (1.0)	1.6 (1.0)	1.3 (1.0)	.01
General health^h^ (fair/poor), no. (%)	113 (48.5)	65 (63.1)	48 (36.9)	<.001
**Comorbidities^i^**				
No. of comorbidities, mean (SD)	1.3 (1.6)	1.6 (1.9)	1.0 (1.3)	.005
High blood pressure, no. (%)	64 (27.5)	35 (34.0)	29 (22.3)	<.001
Heart disease, no. (%)	10 (4.3)	7 (6.8)	3 (2.3)	<.001
Circulation problems, no. (%)	58 (24.9)	33 (32.0)	25 (19.2)	<.001
Stroke, no. (%)	4 (1.7)	1 (1.0)	3 (2.3)	.001
Diabetes, no. (%)	33 (14.2)	23 (22.3)	10 (7.7)	<.001
Depression, no. (%)	45 (19.3)	23 (22.3)	22 (16.9)	<.001

Abbreviation: SD, standard deviation.

a *Camine con Gusto* is a 6-week Spanish-language walking program for adults with arthritis, which participants complete on their own using a workbook.

b χ^2^ test for categorical variables; *t* test for continuous variables.

c Obesity defined as having a body mass index (kg/m^2^) ≥30.0.

d Pain, fatigue, and stiffness were measured by using 10-point visual analogue scales, with 0 being “none” and 10 being “pain/fatigue/stiffness as bad as can be.” Scores were converted to a 100-point scale for analysis.

e The Arthritis Self Efficacy Scale has 11 items that characterize confidence in managing arthritis pain and symptoms. Options range from 1 (very uncertain) to 10 (very certain), with the average of the 11 items used in analysis.

f The Health Assessment Questionnaire measures perceived level of difficulty performing activities of daily living. It has 8 items on common activities, with each item ranging from 0 (without any difficulty) to 3 (unable to do). The average of the 8 items was used in analysis.

g The Rheumatology Attitudes Index is a 5-item subscale that measures perceived helplessness. Each item is scored from 0 to 4 (least to greatest amount of helplessness), and the average was used for analysis.

h Participants were asked to rate their general health as excellent, very good, good, fair, or poor.

i Participants reported each condition they had from a list of 13 common conditions (cancer, fibromyalgia, glaucoma, emphyse ma, high blood pressure, heart disease, circulation problems, diabetes, stomach or intestinal disorders, osteoporosis, chronic liver or kidney disease, stroke, or depression).

About half of all participants rated their general health status as fair or poor, but the group without a high school diploma was significantly more likely to report their general health as fair or poor compared with their more educated counterparts (OR = 2.40; 95% CI, 1.28–4.53) in analyses adjusted for sex, age, obesity, marital status, language spoken, and country of origin (Table 2). They also had a higher mean number of comorbidities (1.6 vs 1.0; *P* = .005) (Table 1) and had greater odds of having more than 2 comorbid conditions, although these odds ratios were not significant (Table 2).

**Table 2:** Association Between Having <High School Diploma^a^ and Health Status Measures at Baseline Among Participants in the *Camine Con Gusto* (Walk With Ease) Program^b^ Who Completed 6-Week Follow-Up, North Carolina, May–September, 2014

Characteristic	<High School Diploma^a^^,^ c, OR (95% CI)	*P* Value
**Self-reported health status**		
Fatigue score^d^ ≥60	1.88 (1.00–3.54)	.05
Pain score^d^ ≥60	1.61 (0.86–3.01)	.14
Stiffness score^d^ ≥60	1.59 (0.87–2.92)	.13
Arthritis Self-Efficacy Scale score^e^ ≤7	1.95 (1.05–3.63)	.04
Health Assessment Questionnaire score^f^ ≥0.5	1.01 (0.55–1.85)	.97
Rheumatology Attitudes Index score^g^ ≥2	1.71 (0.87–3.36)	.12
General health^h^ is fair/poor	2.40 (1.28–4.53)	.01
**Comorbidities^i^**		
No. of comorbidities ≥2	1.08 (0.56–2.08)	.81
High blood pressure	1.37 (0.67–2.78)	.39
Heart disease	3.07 (0.57–16.52)	.19
Circulation problems	1.38 (0.68–2.81)	.37
Stroke	0.22 (0.02–2.64)	.23
Diabetes	2.22 (0.90–5.50)	.08
Depression	1.39 (0.64–3.02)	.40

Abbreviations: CI, confidence interval; OR, odds ratio.

a Compared with having an education ≥high school; data were multiply imputed for missing covariates and predictors.

b *Camine con Gusto* is a 6-week Spanish-language walking program for adults with arthritis, which participants complete on their own using a workbook.

c Adjusted for sex, age, obesity, marital status, language spoken, and country of origin; study site adjusted for as a random effect.

d Pain, fatigue, and stiffness were measured by using 10-point visual analogue scales, with 0 being “none” and 10 being “pain/fatigue/stiffness as bad as can be.” Scores were converted to a 100-point scale for analysis.

e The Arthritis Self Efficacy Scale has 11 items that characterize confidence in managing arthritis pain and symptoms. Options range from 1 (very uncertain) to 10 (very certain), with the average of the 11 items used in analysis.

f The Health Assessment Questionnaire measures perceived level of difficulty performing activities of daily living. It has 8 items on common activities, with each item ranging from 0 (without any difficulty) to 3 (unable to do). The average of the 8 items was used in analysis.

g The Rheumatology Attitudes Index is a 5-item subscale that measures perceived helplessness. Each item is scored from 0 to 4 (least to greatest amount of helplessness), and the average was used for analysis.

h Participants were asked to rate their general health as excellent, very good, good, fair, or poor.

i Participants reported each condition they had from a list of 13 common conditions (cancer, fibromyalgia, glaucoma, emphysema, high blood pressure, heart disease, circulation problems, diabetes, stomach or intestinal disorders, osteoporosis, chronic liver or kidney disease, stroke, or depression).

In measures of arthritis symptoms, the group without a high school diploma was approximately 60% to 90% more likely to report high scores (≥60) for arthritis pain, stiffness, and fatigue at baseline (odds ratios range, 1.59–1.88), although none of these variables were significant in adjusted analyses (Table 2). Surprisingly, the group without a high school diploma had higher mean arthritis symptom scores despite lower rates of obesity. In measures of psychosocial factors, the group without a high school diploma was significantly more likely to report low scores (≤7) for arthritis self-efficacy (OR = 1.95; 95% CI, 1.05–3.63), although not for physical function (Health Assessment Questionnaire score ≥0.5: OR = 1.01; 95% CI, 0.55–1.85) or helplessness (Rheumatology Attitudes Index score ≥2: OR = 1.71; 95% CI, 0.87–3.36).

Both groups improved in arthritis symptoms, physical function, and psychosocial variables at 6-week follow-up (Table 3); therefore, the program seemed to be effective regardless of educational attainment. In fact, the change in primary and secondary outcome measures from baseline to follow-up was slightly greater for most outcome measures in the group without a high school diploma, although none of the effect sizes were significant.

**Table 3:** Baseline and 6-Week Follow-Up Scores, Mean Change Scores^a^, and Effect Sizes^b^ for Participants in *Camine Con Gusto* (Walk With Ease) Program^c^, by Level of Education, North Carolina, May–September, 2014

Outcome Measures	<High School Diploma	≥High School Diploma	Effect Size^b^
**Arthritis Symptoms^d^**			
**Pain**			
No. of respondents	100	127	—
Baseline score, mean (SD)	64.5 (57.3)	58.6 (58.0)	—
Follow-up score, mean (SD)	44.5 (58.5)	41.5 (59.3)	—
Mean change (95% CI) [*P* value]^a ^	−20.0 (−26.1 to −13.9) [<.001]	−17.1 (−22.6 to −11.7) [<.001]	−0.05 (−0.31 to 0.21) [.71]
**Fatigue**			
No. of respondents	99	123	—
Baseline score, mean (SD)	52.3 (65.6)	46.6 (65.7)	—
Follow-up score, mean (SD)	36.4 (64.9)	31.0 (65.0)	—
Mean change (95% CI) [*P* value]^a ^	−15.9 (−22.7 to −9.1) [<.001]	−15.6 (−21.7 to −9.4) [<.001]	0 (−0.26 to 0.27) [.97]
**Stiffness**			
No. of respondents	97	122	—
Baseline score, mean (SD)	40.6 (60.1)	41.8 (60.6)	—
Follow-up score, mean (SD)	27.7 (58.9)	21.9 (59.5)	—
Mean change (95% CI) [*P* value]^a^	−13.0 (−20.0 to −6.0) [<.001]	−19.8 (−26.1 to −13.5) [<.001]	0.11 (−0.15 to 0.38) [.40]
**Physical Function**			
**Health Assessment Questionnaire^e^**			
No. of respondents	99	123	—
Baseline score, mean (SD)	0.5 (1.0)	0.5 (1.0)	—
Follow-up score, mean (SD)	0.3 (1.0)	0.3 (1.0)	—
Mean change (95% CI) [*P* value]^a ^	−0.1 (−0.2 to −0.03) [.01]	−0.19 (−0.28 to −0.10) [<.001]	0.06 (−0.20 to 0.33) [.65]
**Psychosocial Factors**			
**Rheumatology Attitudes Index^f^**			
No. of respondents	97	117	—
Baseline score, mean (SD)	1.5 (1.9)	1.2 (1.8)	—
Follow-up score, mean (SD)	1.3 (1.8)	1.0 (1.8)	—
Mean change (95% CI) [*P* value]^a ^	−0.23 (−0.42 to −0.03) [.02]	−0.23 (−0.41 to −0.05) [.01]	0 (−0.27 to 0.27) [.98]
**Arthritis Self Efficacy^g^**			
No. of respondents	101	121	—
Baseline score, mean (SD)	6.6 (4.5)	7.4 (4.5)	—
Follow-up score, mean (SD)	7.6 (4.5)	8.0 (4.4)	—
Mean change (95% CI) [*P* value]^a ^	0.94 (0.48 to 1.41) [<.001]	0.65 (0.22 to 1.08) [.003]	0.07 (−0.20 to 0.33) [.62]

a We used multivariate linear mixed regression models with recruitment site as a random effects variable to calculate mean changes between baseline and 6-week follow-up scores, controlling for baseline outcome score and covariates (sex, age, marital status, body mass index, language spoken, country of origin, and comorbidities). Analysis included only participants who completed 6-week follow-up. Missing values for variables multiply imputed.

b Mean change scores were used to estimate effect sizes, expressed as Cohen *d*, which was calculated by comparing the mean change scores from baseline to 6 weeks divided by the pooled standard deviation (21).

c *Camine con Gusto* is a 6-week Spanish-language walking program for adults with arthritis, which participants complete on their own using a workbook.

d Pain, fatigue, and stiffness were measured by using 10-point visual analogue scales, with 0 being “none” and 10 being “pain/fatigue/stiffness as bad as can be.” Scores were converted to a 100-point scale for analysis.

e The Health Assessment Questionnaire measures perceived level of difficulty performing activities of daily living. It has 8 items on common activities, with each item ranging from 0 (without any difficulty) to 3 (unable to do). The average of the 8 items was used in analysis.

f The Rheumatology Attitudes Index is a 5-item subscale that measures perceived helplessness. Each item is scored from 0 to 4 (least to greatest amount of helplessness), and the average was used for analysis.

g The Arthritis Self Efficacy Scale has 11 items that characterize confidence in managing arthritis pain and symptoms. Options range from 1 (very uncertain) to 10 (very certain), with the average of the 11 items used in analysis.

Unlike outcome measures, program adherence measures did differ by education. The group without a high school diploma was significantly less likely to have read some or most of the CCG workbook than they were to have read none or a little, in analyses adjusted for sex, age, obesity, marital status, country of origin, and language spoken (OR = 0.51; 95% CI, 0.27–0.97). The group without a high school diploma was also less likely than the group with a high school diploma to report walking 3 or 4 days (OR = 0.58; 95% CI, 0.21–1.55) and 5 or more days (OR = 0.75; 95% CI, 0.28–2.06) per week compared with 1 or 2 days. These odds ratios were not significant in analyses adjusted for sex, age, obesity, marital status, country of origin, and language spoken.

## Discussion

In this study, almost half (44.2%) of participants reported not having a high school diploma. Participants with less than a high school diploma were more likely to report high scores for arthritis pain, fatigue, and stiffness, low levels of arthritis self-efficacy, and poorer general health during baseline assessment. The enhanced burden of arthritis on this group of participants highlights the importance of ensuring that arthritis interventions for Latinos are appropriate for those with less formal educational attainment.

The *Camine Con Gusto* program evaluated in the parent study was a self-directed program based on a workbook. Because engaging with the workbook requires a certain degree of literacy, we expected that the program might be less effective among those without a high school diploma than among those who had graduated from high school. Contrary to expectations, our study showed no differences in primary or secondary measures of program efficacy based on education. However, the group without a high school diploma did report reading less of the workbook compared with the group with the high school diploma. One possible explanation for the lack of difference in follow-up variables is that during recruitment, program staff verbally reviewed the workbook and explained the main objectives of the walking program. This recruitment method may have reduced the need for participants to read the entire workbook to benefit from the program and could explain why no significant differences emerged in the number of days that participants reported walking during the program. Additionally, being involved in a research study may have motivated participants to walk independently of whether or not they read the book. However, the program’s efficacy regardless of educational level suggests that a program dependent on written materials, like CCG, could still hold promise for those with lower educational attainment, especially when paired with another medium of communication, such as a short verbal introduction to the program like the one the recruitment team provided.

The difference in participants’ engagement with the workbook could also suggest a need to adapt the peripheral elements (for example, program information presented in workbook format) of the program so that they better support the core elements (for example, walking). Implementation science emphasizes the importance of implementing evidence-based programs with fidelity to the core components of the program but also allowing for adaptations to peripheral elements to improve the program’s fit within new contexts and populations (22). In our study, the CCG program was effective at improving arthritis outcomes among those with both higher and lower educational attainment — and both groups reported walking similar amounts while participating in the program. Walking may therefore be the most important core element of this program, directly influencing the desired arthritis outcomes. Future research could explore whether the method by which information about walking safely and effectively is conveyed (for example, by book vs in-person communication) could be altered to fit populations with different levels of formal educational attainment.

Our study has several strengths, including a large number of participants and a good distribution of educational levels; the use of validated, participant-reported outcome measures for Latinos; and the inclusion of measures of acculturation (language and birthplace) as covariates in adjusted analyses. Our study also has several limitations. All outcome measures were self-reported, and no performance measures for health status or physical activity were included. In addition, all participants were recruited from the same geographic location in North Carolina.

Our study supports previous research showing that low educational attainment is associated with worse symptoms and health outcomes for Latinos with arthritis (3–5). Although baseline health status differed according to level of education, we found no differences in the extent to which health outcomes changed after the CCG intervention. However, participants without a high school diploma engaged with the program differently than did participants with a high school diploma in that they were less likely to read the program workbook. Given the high percentage of Latinos in the United States that lack a high school diploma, the appropriateness of interventions for populations with low educational attainment should be considered in the process of translation and cultural adaptation.
